# Using genetically isolated populations to understand the genomic basis of disease

**DOI:** 10.1186/s13073-014-0083-5

**Published:** 2014-10-17

**Authors:** Eleftheria Zeggini

**Affiliations:** Wellcome Trust Sanger Institute, Hinxton, Cambridge, CB10 1SA UK

## Abstract

Rare variation has a key role in the genetic etiology of complex traits. Genetically isolated populations have been established as a powerful resource for novel locus discovery and they combine advantageous characteristics that can be leveraged to expedite discovery. Genome-wide genotyping approaches coupled with sequencing efforts have transformed the landscape of disease genomics and highlight the potentially significant contribution of studies in founder populations.

## Complex trait locus discovery in isolated populations

Genetically isolated or founder populations have recently returned to the fore of genetic association studies as valuable resources for complex trait gene identification [1]. Population isolates have well-documented characteristics, including reduced phenotypic, environmental and genetic heterogeneity, that can aid in the detection of rare variants associated with complex traits. In isolated populations, where a relatively small number of individuals found a new population, rare variants that were present in the founders can drift up in frequency as the population expands, thus increasing power for genetic association studies. The small effective population size, which remains small over time, leads to increased levels of homozygosity and linkage disequilibrium. In addition, isolated population cohorts often provide the opportunity to recall subjects by genotype, access detailed genealogical records, obtain linkage to health records and follow the cohort longitudinally.

Recent successes in the literature have highlighted how these advantageous characteristics can help with disease gene mapping. Researchers studying the Icelandic population have, in recent years, pioneered the use of next-generation association studies, a hybrid of genome-wide genotyping and whole genome sequencing (WGS) approaches, for complex disease gene mapping [2,3]. In Iceland, numerous novel loci for complex diseases, such as type 2 diabetes (T2D) and prostate cancer [4,5], have been identified through a combination of WGS and long-range phasing-assisted imputation on a genome-wide genotype scaffold, together with calculation of genotype probabilities in approximately 300,000 untyped individuals by making use of the extended genealogical information available.

More recently, novel insights into the biological pathways underpinning T2D were achieved through the study of a Greenlandic founder population [6]. A nonsense variant in the *TBC1D4* gene was found to be strongly associated with postprandial hyperglycemia, impaired glucose tolerance and T2D. These unique insights into the mechanism conferring muscle insulin resistance for this subset of T2D was afforded by studying the small Greenlandic population, which has experienced a dramatic increase in T2D prevalence, and recalling individuals based on their *TBC1D4* variant status. This polymorphism is common in Greenland (17% minor allele frequency), but vanishingly rare in other global populations (only encountered in one Japanese individual in the 1000 Genomes Project data). This work elegantly demonstrates the value of combining the genetic characteristics of founder populations with the potential to recontact participants for further follow-up of promising results. Studies in extensively phenotyped founder population cohorts, such as the Amish, have also demonstrated the value of combining unique population characteristics with recall of subjects to increase our understanding of disease etiopathology. The Old Order Amish are a cultural isolate and geographically localized, genetically homogeneous population with extensive genealogical records available. This deeply phenotyped cohort has been the subject of long-term genetic studies. For example, in 2008, Pollin *et al.* [7] reported a missense variant (R19X) that abolishes expression of the *APOC3* gene and is strongly associated with a cardioprotective phenotype (higher high-density lipoprotein and lower blood triglyceride levels).

Notably, the same missense cardioprotective variant was also found in an independent isolated population from Greece in the HELIC-MANOLIS study [8]. Residents of the mountainous Mylopotamos villages on Crete have a high fat content diet but anecdotally display lower levels of, for example, T2D complications compared with the general population. The R19X *APOC3* variant was carried by approximately 4% of the individuals studied and reached genome-wide statistical significance with a sample size of fewer than 1,300. Discovery of the same effect in the general population would have required over 50 times the number of subjects. Large-scale studies of over 110,000 individuals of European descent have recently also established an association of rare variants in the *APOC3* locus with protection against high triglyceride levels and coronary artery disease [9]. *APOC3* is now becoming a poster child for the power afforded by founder populations and clearly demonstrates the generalizability of findings in isolates into more cosmopolitan populations.

A prime example of how founder population characteristics coupled with linkage to medical records can accelerate discovery was recently produced by studying the Finnish population [10]. In a whole exome sequencing study of about 3,000 Finns, Lim *et al.* first established that the Finns have fewer variable sites overall but more loss-of-function variants compared with non-Finnish European individuals, and subsequently identified robust associations with key traits of medical relevance. Linkage to national medical records resulted in the demonstration that splice variants in the *LPA* gene that are associated with low levels of plasma lipoprotein(a) confer protection against cardiovascular disease.

## Future directions

Going forward, it is clear that founder populations can provide a unique and powerful resource for the identification of low frequency and rare variants of direct medical consequence. Power to detect association is demonstrably boosted for individual sequence variants that have drifted up in frequency. In addition, power to detect a significant accumulation of rare variants at particular loci is further increased in founder populations as neutral rare variation may be lost from the haplotype pool. In this context, meta-analysis at the locus level across different isolates is posited to be important for establishing burden of proof, although this principle requires empirical substantiation. Historically, the transferability of findings in isolates across to more cosmopolitan populations has been a topic of debate. However, there is an accrual of emerging examples of loci discovered in founder populations that are more widely generalizable, with replication of signals achieved in diverse sample sets [4,5,7-9]. Furthermore, invaluable and unprecedented insights into disease pathogenesis can be afforded by findings restricted to genetically isolated populations, as exemplified by the elegant metabolic trait study in Greenland [6]. Decreasing costs for deep whole genome sequencing and the increasing availability of deeply phenotyped genetically isolated cohorts sets the scene for further success stories in the near future.

## Abbreviations

T2D: Type 2 diabetes
WGS: Whole genome sequencing
